# Interferon-Gamma DNA Methylation Is Affected by Mycophenolic Acid but Not by Tacrolimus after T-Cell Activation

**DOI:** 10.3389/fimmu.2017.00822

**Published:** 2017-07-12

**Authors:** Fleur S. Peters, Annemiek M. A. Peeters, Leo J. Hofland, Michiel G. H. Betjes, Karin Boer, Carla C. Baan

**Affiliations:** ^1^Nephrology and Transplantation, Department of Internal Medicine, Erasmus University Medical Center Rotterdam, Erasmus MC, Rotterdam, Netherlands; ^2^Endocrinology, Department of Internal Medicine, Erasmus University Medical Center Rotterdam, Erasmus MC, Rotterdam, Netherlands

**Keywords:** interferon-gamma, epigenetics, polyclonal activation, remethylation, transplantation immunology, *in vitro*

## Abstract

Immunosuppressive drug therapy is required to treat patients with autoimmune disease and patients who have undergone organ transplantation. The main targets of the immunosuppressive drugs tacrolimus and mycophenolic acid (MPA; the active metabolite of mycophenolate mofetil) are T cells. It is currently unknown whether these immunosuppressive drugs have an effect on DNA methylation—an epigenetic regulator of cellular function. Here, we determined the effect of tacrolimus and MPA on DNA methylation of the gene promoter region of interferon gamma (IFNγ), a pro-inflammatory cytokine. Total T cells, naive T cells (CCR7^+^CD45RO^−^), and memory T cells (CD45RO^+^ and CCR7^−^CD45RO^−^) were isolated from CMV seropositive healthy controls and stimulated with α-CD3/CD28 in the presence or absence of tacrolimus or MPA. DNA methylation of the *IFN*γ promoter region was quantified by pyrosequencing at 4 h, days 1, 3, and 4 after stimulation. In parallel, T-cell differentiation, and IFNγ protein production were analyzed by flow cytometry at days 1 and 3 after stimulation. Our results show that MPA induced changes in *IFN*γ DNA methylation of naive T cells; MPA counteracted the decrease in methylation after stimulation. Tacrolimus did not affect *IFN*γ DNA methylation of naive T cells. In the memory T cells, both immunosuppressive drugs did not affect *IFN*γ DNA methylation. Differentiation of naive T cells into a central-memory-like phenotype (CD45RO^+^) was inhibited by both immunosuppressive drugs, while differentiation of memory T cells remained unaffected by both MPA and tacrolimus. IFNγ protein production was suppressed by tacrolimus. Our results demonstrate that MPA influenced *IFN*γ DNA methylation of naive T cells after stimulation of T cells, while tacrolimus had no effect. Both tacrolimus and MPA did not affect *IFN*γ DNA methylation of memory T cells.

## Introduction

Patients who have undergone organ transplantation as well as patients with autoimmune disease require lifelong immunosuppression to inhibit the immune response toward alloantigen or autoantigen. This immune response involves interaction between different immune cells including dendritic cells, macrophages, T, and B cells. T cells proliferate, differentiate, and produce effector cytokines in response to antigen (1, 2) and therefore immunosuppressive drugs are often designed to suppress T-cell activity.

After activation, the differentiation of T cells is regulated to great extent by DNA methylation—an essential epigenetic regulator of several cellular functions (3–5). DNA methylation is the addition of a methyl group on a cytosine (C) that is followed by a guanine (G) in the DNA, also known as a CpG dinucleotide. High methylation in the promoter region of a gene is related to a closed chromatin structure and transcriptional silencing of the gene (6, 7). When T cells differentiate during an immune response, the promoter regions of various effector genes become demethylated, thereby allowing the cells to upregulate these genes and produce effector cytokines (8, 9). Naive T cells are therefore characterized by methylated promoter regions of effector genes, whereas effector and memory T cells are demethylated at those regions.

Epigenetic regulators such as DNA methylation are dynamic and susceptible to cues from the environment (10, 11). These cues include internal factors such as cytokines and hormones as well as external factors such as food, toxins, and drugs. Several common-used pharmaceutical drugs, not designed as epigenetic drugs, have an effect on epigenetic mechanisms in the cell (12, 13). These findings suggest that immunosuppressive drugs could affect DNA methylation in T cells and thereby modulate T-cell function.

Today, the immunosuppressive drugs that are most often prescribed to organ transplant recipients include tacrolimus and mycophenolate mofetil (14, 15). Tacrolimus represses the calcineurin pathway downstream of the T-cell receptor. It inhibits calcineurin phosphatase activity, thereby reducing levels of dephosphorylated nuclear factor of activated T (NFAT) lymphocytes, which ultimately inhibits T-cell activation (16, 17). Mycophenolate mofetil’s active ingredient is mycophenolic acid (MPA). MPA is an inhibitor of inosine monophosphate dehydrogenase (IMPDH), a key enzyme in *de novo* purine synthesis (18). Inhibition of IMPDH reduces synthesis of guanosine nucleotides, which are essential for DNA synthesis in T cells, resulting in reduced proliferation of T cells (19, 20). Despite the fact that the mechanism of action is largely known for these two drugs, it is not known whether their effect on cellular function involves epigenetic regulation, or whether they affect the epigenetic regulation of cytokine expression. A further understanding of the effect of different immunosuppressive drugs on epigenetic regulators of T-cell function will contribute to optimization of the immunosuppressive regimen.

We hypothesized that tacrolimus and MPA induce changes in DNA methylation of T cells. We focus on promoter DNA methylation of the pro-inflammatory cytokine interferon gamma (IFNγ) which plays a prominent role in immune responses. Not only have high expression levels of IFNγ been linked to acute rejection after organ transplantation (21–23), it is also highly expressed during the inflammation seen in autoimmunity (24, 25). IFNγ expression—along with that of many other cytokines—is known to be regulated by DNA methylation (26–28). To study the effect of immunosuppressive drugs on *IFN*γ DNA methylation after activation of T cells, we stimulated T cells *in vitro* in the absence or presence of tacrolimus or MPA. After stimulation, DNA methylation was measured at two sites within the *IFN*γ promoter. Since DNA methylation is cell-type specific (29), the experiments were performed on total T cells as well as on isolated naive and memory T cells.

## Materials and Methods

### Study Subjects

Our study population consisted of 19 healthy individuals aged between 26 and 75 (68% female). Peripheral blood of these subjects was collected after informed consent and according to biobank protocol with approval of the local ethics committee (MEC-2010-022). We chose to study healthy individuals to eliminate confounding effects of disease on DNA methylation (30). It is also known that *IFN*γ DNA methylation is significantly lower in CMV seropositive individuals than in CMV seronegative individuals (31). To compose a homogeneous group and eliminate CMV effects on inter-individual differences in methylation levels, only CMV seropositive individuals were included in the study.

### Isolation of Total T Cells, Naive T Cells, and Memory T Cells

Peripheral blood mononuclear cells (PBMCs) were isolated from the peripheral blood by density gradient centrifugation using Ficoll-Paque (GE Healthcare, Chicago, IL, USA). Isolated PBMCs were stored at −140°C until further use. Total T cells were isolated from the PBMCs by magnetic cell separation on the autoMACS (Miltenyi Biotech, Bergisch Gladbach, Germany) according to the pan T cell protocol using the deplete S settings. Purities were >90% CD3^+^ cells after isolation.

The naive and memory T-cell populations were isolated from the PBMCs using fluorescence-activated cell sorting (FACS) by the BD FACSAria™ II (BD Biosciences, San Jose, CA, USA). The PBMCs were stained with CD3 Brilliant Violet 510 (Biolegend, San Diego, CA, USA), CD4 Pacific Blue (BD Biosciences), CD8 APC-cy7 (BD Biosciences), CD45RO APC (Biolegend), CCR7 PE-cy7 (BD Biosciences), and to exclude non-viable cells the cells were also stained with 7AAD PerCP (BD Biosciences). Naive cells were defined as CCR7^+^CD45RO^−^, central memory cells as CCR7^+^CD45RO^+^, effector memory (EM) as CCR7^−^CD45RO^+^, and the highly differentiated EMRA cells as CCR7^−^CD45RO^−^ (32). After cell sorting, the purities were >95% for each sorted fraction.

### T-Cell Stimulation

The T cells were stimulated for 4 days with α-CD3/CD28 coated Dynabeads^®^ (Gibco, Waltham, MA, USA) in a bead to cell ratio of 1:1 at day 0. Fifty thousand cells were cultured per well in a 96-well plate. The cells were cultured in the absence or presence of tacrolimus, MPA or 5-aza-2′deoxycytidine (decitabine). Tacrolimus (Prograf^®^, Astellas Pharma, Tokyo, Japan) was added to the cells in a concentration of 10 ng/mL which is a clinically relevant concentration that is reached in transplant recipients (33). MPA (Sigma-Aldrich, St. Louis, MO, USA) was added to the cells in a concentration of 0.2 µg/mL, a concentration at which the cells are still able to proliferate. Our positive control, the demethylating agent decitabine (Sigma-Aldrich) (34), was added to the cells in a concentration of 10^−6^ M, a concentration at which the cells are still able to proliferate. Each drug-treated sample has a matched negative control (stimulation alone).

The cells were incubated at 37°C in 5% CO_2_ and harvested at 4 h, days 1, 3, and 4 for DNA methylation analysis, and at days 1 and 3 for flow cytometry analysis. To assess viability and proliferation, the cells were counted before and after stimulation using conventional light microscopy and Trypan Blue staining (Thermo Fisher Scientific, Waltham, MA, USA).

### Flow Cytometry

Flow cytometry was used to determine the phenotype of T cells immediately after isolation and at days 1 and 3 after stimulation. We also measured the percentage of IFNγ producing cells at these time points. The samples were treated with Brefeldin A (GolgiPlug™, BD Biosciences) for 16 h prior to flow cytometry analysis. The monoclonal antibodies used for cell surface staining were the same as previously described for the FACS cell sorting. In addition, the cells were permeabilized using permeabilize solution 2 (BD Biosciences), and stained for intracellular IFNγ with FITC labeled IFNγ (BD Biosciences). The cells were then analyzed on the FACSCanto II (BD Biosciences) with FACSDiva software. All flow cytometry data were analyzed using Kaluza software 1.3 (Beckman Coulter, Brea, CA, USA).

### DNA Isolation, Bisulfite Conversion, and PCR

After harvesting, the cells they were pelleted, frozen in liquid nitrogen, and stored at −80°C until bisulfite conversion. The T-cell pellets were digested with proteinase K and bisulfite treatment was performed using the EZ DNA Methylation-Direct kit (Zymo Research, Irvine, CA, USA) according to the manufacturer’s protocol. Bisulfite treatment introduces methylation-dependent changes in the DNA, demethylated cytosines are converted into uracil whereas methylated cytosines remain unchanged. The bisulfite-treated DNA was amplified by PCR. A 230 base pair region of the *IFN*γ promoter was amplified using the Pyromark PCR kit (Qiagen, Venlo, The Netherlands). A forward primer with the sequence 5′-ATGGTATAGGTGGGTATAATGG-3′ and a biotin-labeled reverse primer with the sequence 5′-CAATATACTACACCTCCTCTAACTAC-3′ (Sigma-Aldrich) were used, both at a concentration of 10 pmol/μL (31). The PCR conditions were 15 min at 95°C, 45 cycles of 30 s 94°C, 30 s 58°C, 30 s 72°C followed by 10 min at 72°C, and final storage at room temperature (21°C). Prior to pyrosequencing, the PCR product was visualized on a 1% agarose gel to verify the size of the amplicon. Two important CpG sites are inside this amplicon, CpG -186 and CpG -54. These sites are within binding domains of transcription factors (26, 31).

### Pyrosequencing

Pyrosequencing is an excellent technique to quantitatively measure DNA methylation at single CpG-site resolution, yielding accurate, and reproducible results (35, 36). The *IFN*γ PCR product was sequenced using a PyroMark Q24 pyrosequencer (Qiagen). Minor adjustments were made to the manufacturer’s protocol: to immobilize the PCR product 1 µL Streptadivin Sepharose High Performance Beads (GE Healthcare) was used per sequence reaction and annealing of the sequence primers was done for 3 min at 80°C. The CpG -186 sequence primer was 5′-GGTGGGTATAATGGG-3′ and the CpG -54 sequence primer was 5′-ATTATTTTATTTTAAAAAATTTGTG-3′, both at a concentration of 10 µM (31). Two DNA methylation standards were used as control, human high, and low methylated DNA (EpigenDx, Hopkinton, MA, USA). Research shows that methylation at adjacent sites is correlated (37) therefore the methylation percentages of the two CpG sites, site -54 and -186, were pooled per individual and the mean DNA methylation percentage is presented in the results.

### Statistical Analysis

Statistical analyses were performed with SPSS Statistics version 21.0 (IBM Corp., Armonk, NY, USA). The Mann–Whitney *U* test was used for unpaired analysis to identify differences between the conditions at a certain time point. The Wilcoxon signed-rank test was used for paired analysis when comparing different time points within a condition. A *p*-value <0.05 was considered statistically significant.

## Results

### Effect of Tacrolimus and MPA on *IFN*γ DNA Methylation of Total T Cells

To exclude complete cell cycle arrest as a cause for methylation differences, we compared cell numbers under the different conditions after stimulation. Cell numbers were lower if cells were cultured with either tacrolimus, MPA, or decitabine than if the cells were cultured without those factors, but due to overlapping ranges this difference was not statistically significant (Figure S1 in Supplementary Material). Our results suggest that the cells were still able to proliferate under the chosen concentrations of the different drugs.

**Figure 1:**
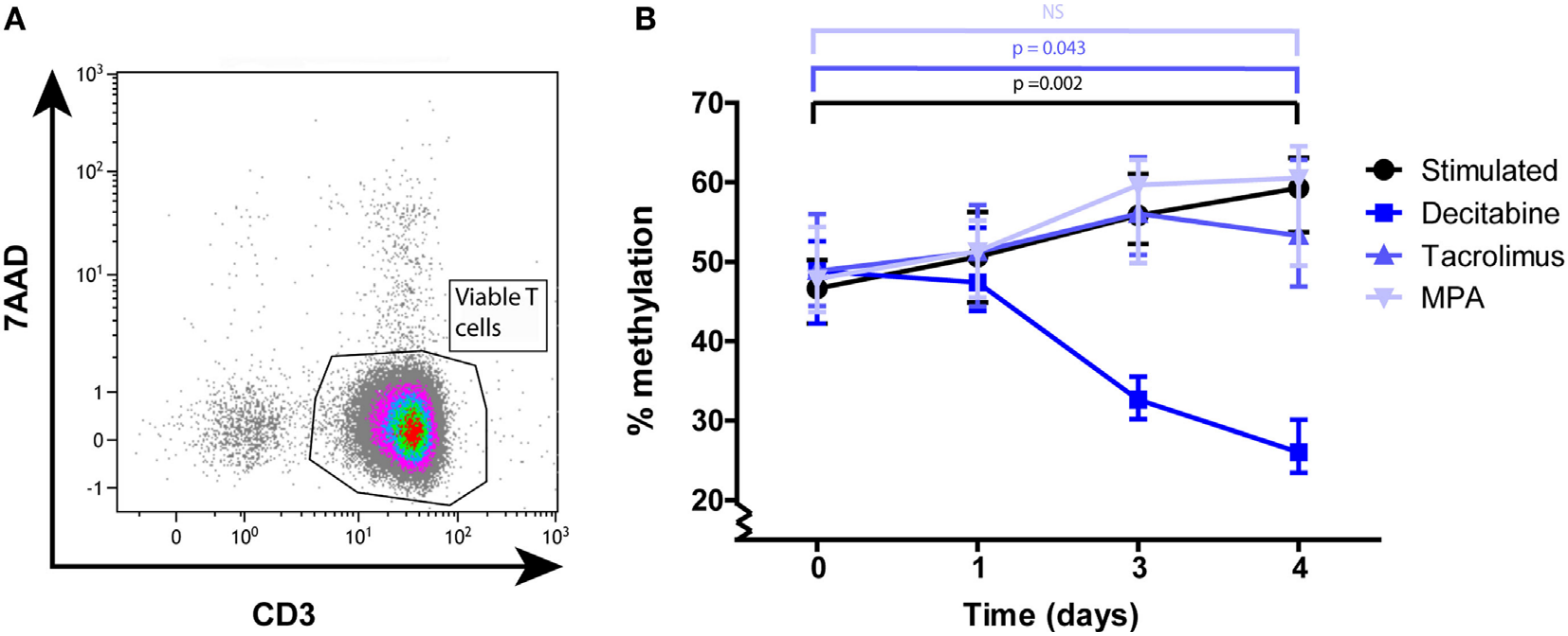
**(A)** A representative example of the CD3^+^ purity and viability after MACS isolation. **(B)** Median and interquartile range of *IFN*γ DNA methylation at days 0, 1, 3, and 4 after α-CD3/CD28 stimulation of total T cells under the different culture conditions: stimulated (*n* = 15), decitabine (*n* = 7), tacrolimus (*n* = 5), mycophenolic acid (*n* = 4). *p*-Values were calculated with a Wilcoxon matched pairs test.

To determine the changes in DNA methylation after T-cell stimulation, we analyzed *IFN*γ promoter methylation at several time points after stimulation. *IFN*γ DNA methylation of total T cells increased significantly after stimulation with α-CD3/CD28 (*p* = 0.002; Figure 1B). Stimulated T cells showed a median DNA methylation percentage of 47% (range: 35–59%) at day 0 and this was significantly increased at day 4 (59%; 46–66%).

DNA methylation of T cells cultured in the presence of tacrolimus increased significantly from 49 (42–59%) to 53% (44–67%) (*p* = 0.043) and did not differ significantly from the stimulated condition at any of the given time points (Figure 1B). DNA methylation of T cells cultured in the presence of MPA increased from 48 (43–56%) to 61% (46–66%) and also did not differ significantly from the stimulated condition (Figure 1B). Our positive control, T cells cultured in the presence of decitabine, significantly decreased in DNA methylation between day 0 and day 4 (*p* = 0.028; Figure 1B).

Since our total T-cell population was a heterogeneous mixture of naive and memory T cells with different methylation profiles (29), we continued to study isolated cell populations to infer whether tacrolimus or MPA did influence these cell types individually.

### Effect of Tacrolimus and MPA on IFNγ DNA Methylation of Naive and Memory T Cells

**Figure 2:**
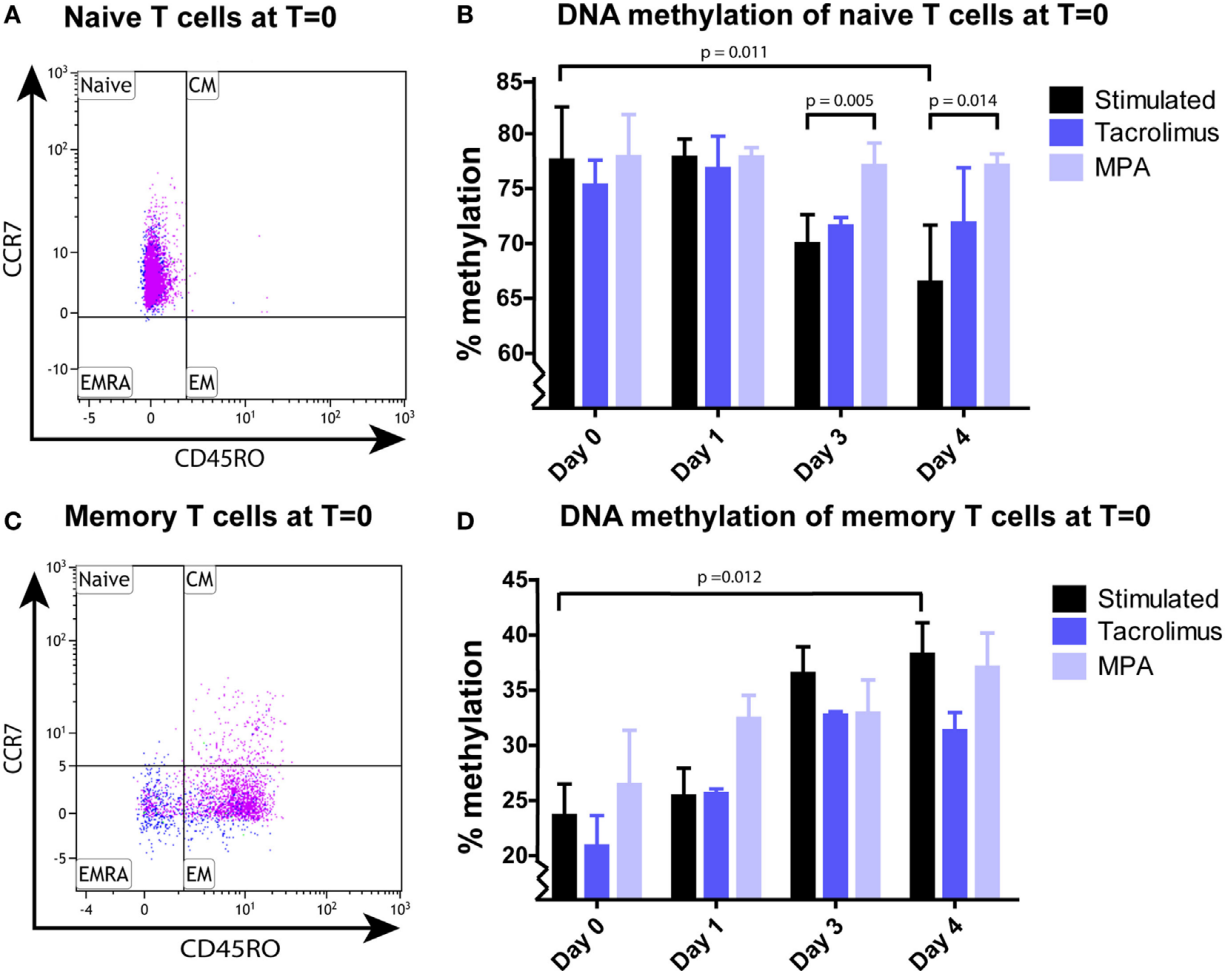
**(A)** A representative example of the naive CCR7^+^CD45RO^−^ T cells after sorting. **(B)** Median and interquartile range of *IFN*γ DNA methylation of sorted naive T cells stimulated in the absence (*n* = 9) or presence of tacrolimus (*n* = 3) or mycophenolic acid (MPA) (*n* = 4). **(C)** A representative example of the memory CD45RO^+^ and CCR7^−^CD45RO^−^ T cells after sorting. **(D)** Median and interquartile range of *IFN*γ DNA methylation of the sorted memory T cells stimulated in the absence (*n* = 9) or presence of tacrolimus (*n* = 3) or MPA (*n* = 3). The pink dots in the fluorescence-activated cell sorting plots **(A,C)** represent the CD4^+^ cells and the blue dots the CD8^+^ cells. *p*-Values were calculated with a Wilcoxon matched pairs test (*T* = 0 vs *T* = 3 within one condition) or Mann–Whitney *U* test (between conditions).

Pure naive (CCR7^+^CD45RO^−^) (Figure 2A) and memory (CD45RO^+^ and CCR7^−^CD45RO^−^) (Figure 2C) T-cell subsets were stimulated separately. *IFN*γ DNA methylation significantly decreased in the naive start population in the absence of tacrolimus or MPA, from 78 (75–83%) at day 0 to 67% (61–77%) at day 4 (*p* = 0.011; Figure 2B). The two immunosuppressive drugs had differential effects on this reduction in DNA methylation. While tacrolimus had no effect, MPA neutralized the effect of stimulation significantly and DNA methylation did not decrease (78%; 76–82% at day 0 and 77%; 75–78% at day 4). This differential effect resulted in a significant difference between stimulation only and the addition of MPA on day 3 (*p* = 0.005) and day 4 (*p* = 0.014; Figure 2B).

In the total memory start population, *IFN*γ DNA methylation significantly increased in the absence of tacrolimus or MPA, from 24 (19–31%) at day 0 to 38% (30–46%) at day 4 (*p* = 0.012; Figure 2D). This increase was not affected by tacrolimus nor MPA, both these conditions were not significantly different from stimulation alone.

**Figure 3:**
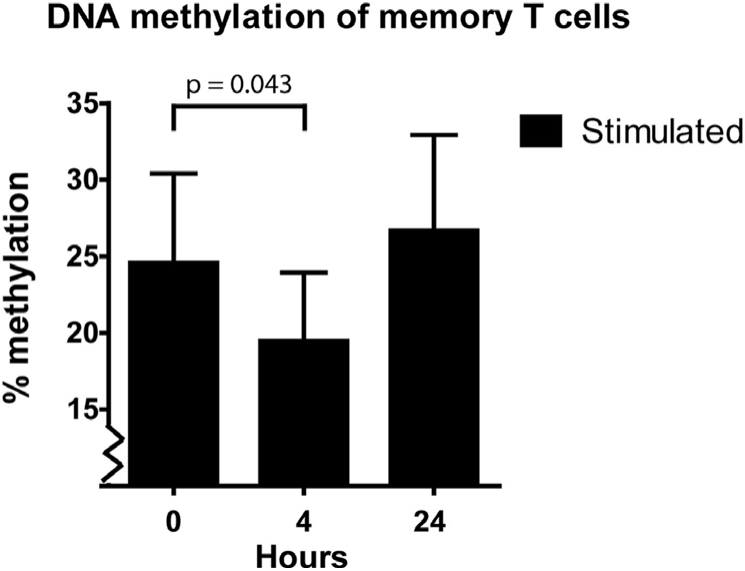
Median and interquartile range of *IFN*γ DNA methylation of the sorted memory T cells at 0, 4, and 24 h after α-CD3/CD28 stimulation (*n* = 5). *p*-value was calculated with a Wilcoxon matched pairs test.

As explained in the Section “Introduction,” we expected effector-gene promoters to demethylate after activation to allow transcription of the corresponding effector gene. We observed this in the naive T cells, demethylation of the *IFN*γ promoter took place after 3 days of stimulation (Figure 2B). However, the *IFN*γ promoter of the memory T cells did not demethylate after 1, 3, or 4 days after stimulation (Figure 2D). Therefore, we speculated that demethylation occurred in a shorter timeframe than 24 h, to allow memory T cells to produce IFNγ protein. To address this question, we harvested memory T cells at 4 h after stimulation and indeed we observed a significant decrease (3–12%; *p* = 0.043) in methylation followed by remethylation to base levels after 24 h (Figure 3).

### Phenotypic Changes after α-CD3/CD28 Stimulation of the Naive T Cells

**Figure 4:**
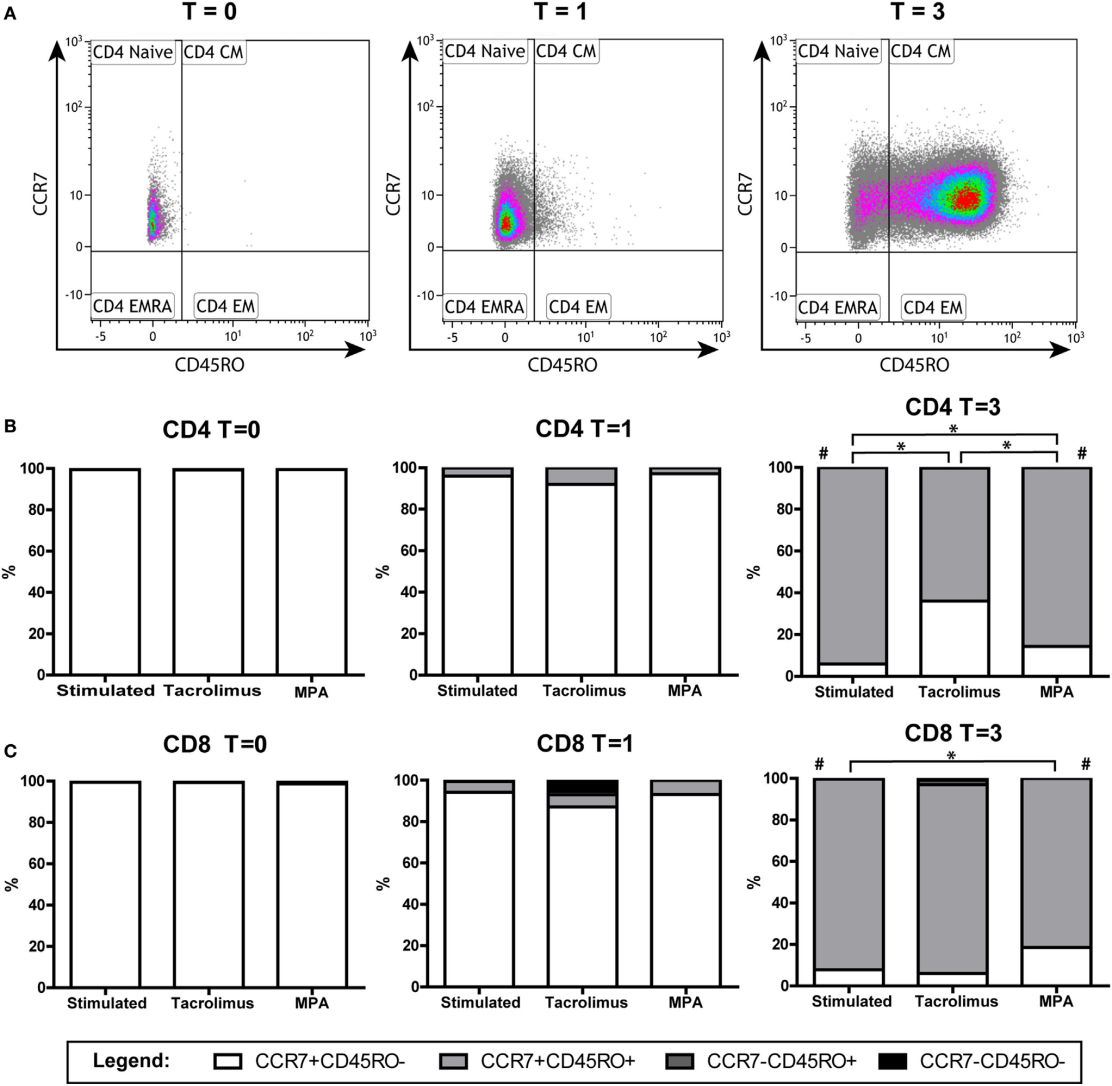
Phenotypic changes of the naive T cells in the absence or presence of tacrolimus or mycophenolic acid (MPA): stimulated (*n* = 9), tacrolimus (*n* = 3), and MPA (*n* = 4). **(A)** A representative gating example of the CD4^+^ T cells directly after isolation (*T* = 0) and at day 1 (*T* = 1) and day 3 (*T* = 3) after stimulation. **(B)** Median percentages of CD4^+^ subsets in the absence or presence of tacrolimus or MPA at days 0, 1, and 3. **(C)** Median percentages of CD8^+^ subsets in the absence or presence of tacrolimus or MPA at days 0, 1, and 3. *p < 0.05 (Mann–Whitney *U* test to compare two conditions); ^#^*p* < 0.05 (Wilcoxon matched pairs test to compare *T* = 0 with *T* = 3 within one condition).

The isolated naive T cells, which were CCR7^+^CD45RO^−^ at day 0, were analyzed for the expression of CD45RO and CCR7 after 1 and 3 days of stimulation in the absence and presence of tacrolimus or MPA. CD4^+^ and CD8^+^ T cells were gated separately (Figure 4), the percentages CD4^+^/CD8^+^ do not differ significantly between the conditions (Figure S2 in Supplementary Material). After 1 day of stimulation, the phenotype did not differ significantly from day 0 in both CD4^+^ and CD8^+^ T cells. On day 3, there was a significant shift toward CD45RO^+^ cells in the stimulated condition (*p* = 0.008). The shift was observed in all three conditions and in both the CD4^+^ and CD8^+^ T cells (Figures 4B,C). These cells, which were CD45RO^−^ at day 0, upregulated their CD45RO expression showing a central-memory-like phenotype at day 3. When we compared the different conditions with stimulation only at day 3, tacrolimus (*p* = 0.013) and MPA (*p* = 0.039) significantly repressed CD4^+^ differentiation and MPA also significantly repressed CD8^+^ differentiation (*p* = 0.014; Figures 4B,C).

### Phenotypic Changes after α-CD3/CD28 Stimulation of the Memory T Cells

**Figure 5:**
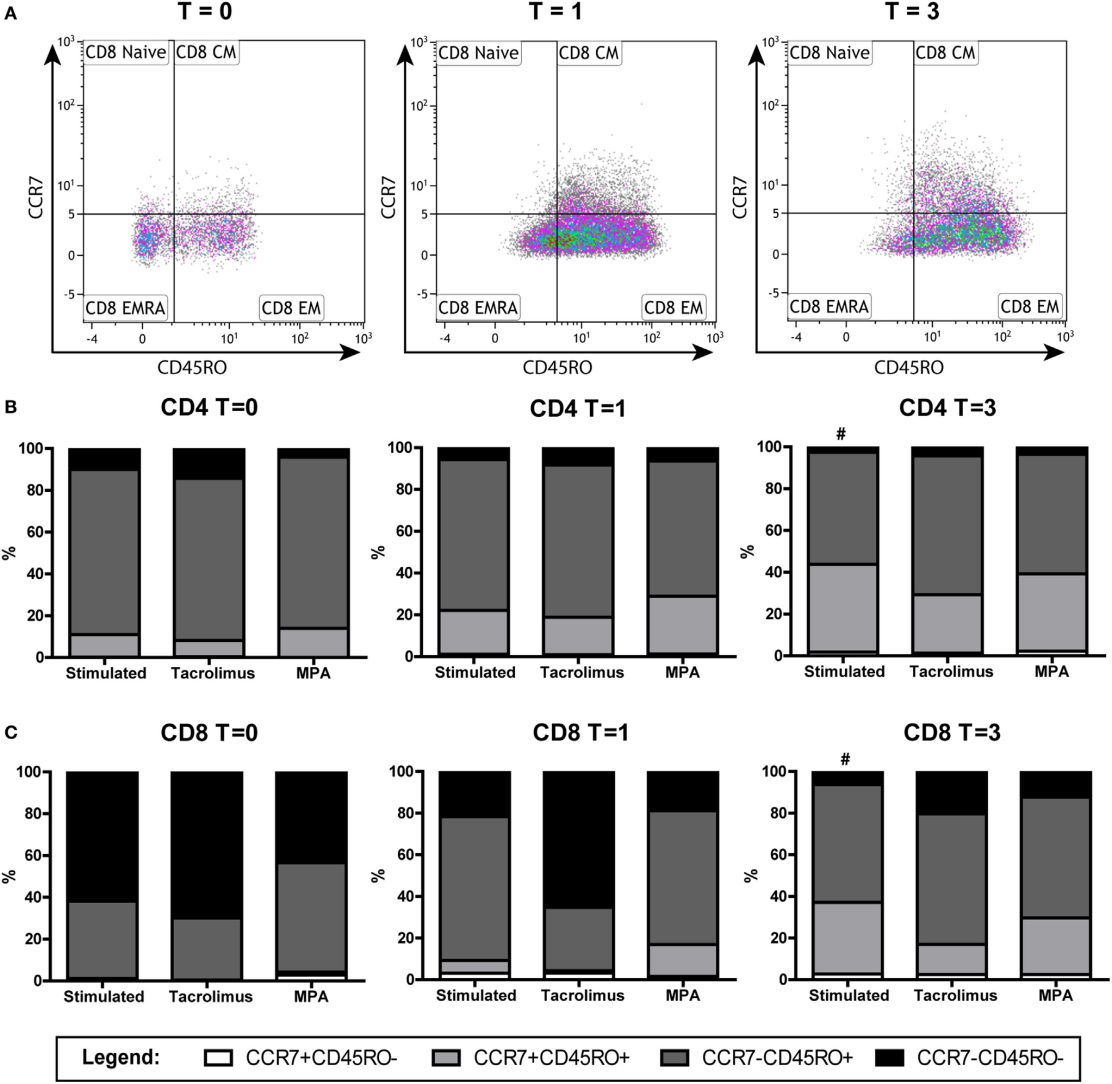
Phenotypic changes of the memory T cells in the absence or presence of tacrolimus or mycophenolic acid (MPA): stimulated (*n* = 9), tacrolimus (*n* = 3), and MPA (*n* = 3). **(A)** A representative gating example of the CD8^+^ subsets of the stimulated cells directly after isolation (*T* = 0) at day 1 (*T* = 1) and day 3 (*T* = 3) after stimulation. **(B)** Median percentages of CD4^+^ subsets in the absence or presence of tacrolimus or MPA at days 0, 1, and 3. **(C)** Median percentages of CD8^+^ in the absence or presence of tacrolimus or MPA at days 0, 1, and 3. ^#^*p* < 0.05 (Wilcoxon matched pairs test to compare *T* = 0 with *T* = 3 within one condition).

The isolated memory T cells, which were CD45RO^+^ and CCR7^−^CD45RO^−^ at day 0, were also analyzed by flow cytometry after 1 and 3 days of stimulation in the absence or presence of tacrolimus or MPA. CD4^+^ and CD8^+^ T cells were gated separately (Figure 5). The percentage of CD8^+^CD45RO^+^ cells increased significantly after 3 days of stimulation, both in the CCR7^+^ (*p* = 0.008) and CCR7^−^ (*p* = 0.021) population (Figure 5C). In the CD4^+^ population, we observed an increase in the CCR7^+^CD45RO^+^ population (*p* = 0.011) and a decrease in the CCR7^−^ population (*p* = 0.021) (Figure 5B). When we compared the different conditions with stimulation only at day 3, no significant differences were found.

### IFNγ Protein Production of the Memory Population

**Figure 6:**
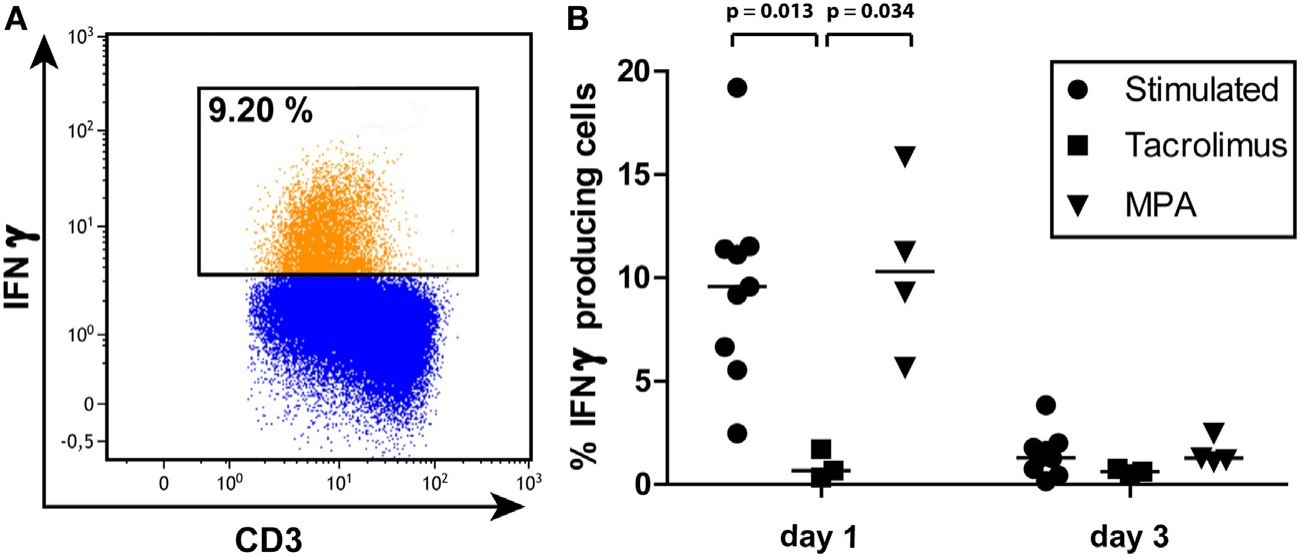
**(A)** A representative gating example of interferon gamma (IFNγ) production by the sorted memory T-cell population on day 1 after stimulation. **(B)** Percentages and median of IFNγ producing memory T cells on days 1 and 3 of all three conditions measured by intracellular staining and flow cytometry. *p*-Values were obtained with the Mann–Whitney *U* test.

Interferon gamma protein production was measured using intracellular staining in both the sorted naive T cells and the sorted memory T cells (Figure 6). The sorted naive T cells did not produce IFNγ protein at day 1 after stimulation (data not shown) while 10% (3–19%) of the sorted memory T cells did produce IFNγ. Tacrolimus significantly inhibited IFNγ production, hardly any cells produced IFNγ in the presence of tacrolimus (Figure 6B). MPA did not have a significant effect on IFNγ production and the percentage IFNγ producing cells did not differ from stimulation only. Three days after stimulation of the sorted memory T cells, few cells still produce IFNγ both in the presence and absence of tacrolimus or MPA.

## Discussion

To our knowledge, this is the first study to investigate the effect of immunosuppressive medication on DNA methylation of primary T cells (38, 39). The study design allowed us to track changes over time after activation. Also, by combining the results of our analyses of DNA methylation, phenotype, and protein production, we were able to determine the effects of immunosuppressive drugs on cellular dynamics after T-cell activation. Our results show that after T-cell activation, MPA affected *IFN*γ DNA methylation of naive T cells but not that of memory T cells, while tacrolimus had no effect on *IFN*γ DNA methylation of T cells (Figures 1 and 2).

The mechanism by which MPA counteracts the effect of T-cell stimulation on *IFN*γ DNA methylation is unknown. We can however suggest a possible mechanism by looking at the different enzymes that regulate DNA methylation in general. DNA methyl transferases (DNMTs) are a family of enzymes that maintain DNA methylation during cell division (DNMT1) and cause *de novo* DNA methylation (DNMT3a,b) (4). Lower activity of DNMT1 leads to passive demethylation, the methylation “dilutes” during cell division (5, 40). Possibly, MPA has a direct or indirect effect on DNMT1 activity during differentiation of naive T cells. A similar suggestion was made by He et al. (41) in relation to an increased CD70 expression induced by MPA.

While the two drugs’ effects on DNA methylation were different, their effects on T-cell differentiation were similar (Figures 4 and 5). Tacrolimus and MPA both suppressed the differentiation of naive T cells (CD45RO^−^) toward CD45RO^+^ cells. This phenotypic marker is a characteristic marker for memory T cells (32) but it has been described as an activation marker as well (42, 43). Since tacrolimus inhibited differentiation of the naive T cells significantly but did not influence *IFN*γ DNA methylation of those cells, we believe that the differentiation can occur independently from changes in *IFN*γ DNA methylation. On the other hand, the changes in T-cell phenotype and *IFN*γ DNA methylation after stimulation alone both occur after 3 days, indicating a relation between these two parameters. Taken together, the exact relationship between phenotypic changes and changes in *IFN*γ DNA methylation after stimulation remains unclear.

While we had expected T cells to become demethylated on their *IFN*γ promoter upon stimulation, we were surprised to note that, in both total T cells and memory T cells, *IFN*γ promoter methylation actually increased (Figures 1B and 2D). In line with the results of previous studies (44, 45), *IFN*γ DNA methylation decreased shortly after stimulation of the memory T cells (Figure 3). After the demethylation phase of these cells, *IFN*γ DNA methylation returned to base-level and from day 1 onward DNA methylation steadily increased. Since the phenotype of the cells changed after stimulation, each time point reflected a heterogeneous cell population. This makes it difficult to assign the increasing *IFN*γ DNA methylation to a specific cell type. The ideal situation would be to isolate pure cell populations at each time point using surface markers before analyzing their methylation profile—this is practically challenging however.

We are currently uncertain what the biological reason is behind the increase in *IFN*γ DNA methylation (remethylation) that we observed. Similar remethylation of gene promoters after stimulation has thus far been reported for PD1 and IL2. Youngblood et al. (46) studied the *PD1* locus in antigen-specific CD8^+^ T cells in mice and found that after 8 days of LCMV infection, the *PD1* locus in effector cells had been partially remethylated. This finding was only seen in an acute infection model however: when the mice were chronically infected, the locus remained demethylated and the CD8^+^ cells became exhausted (46). A study on *IL2* promoter DNA methylation in HIV-infected patients showed that *IL2* DNA methylation was higher in all CD4^+^ EM subsets of HIV-infected patients than in those of healthy controls, indicating that chronic HIV infection increased methylation levels in these cell types (47). The remethylation of the *IFN*γ promoter that we observed may be similar to that of the *PD1* and *IL2* promoters described in the above-mentioned papers.

Although DNA methylation of *IFNy* was not affected by the presence of tacrolimus, IFNγ protein production by the memory cells was suppressed in the presence of tacrolimus (Figure 6). As mentioned in the Section “Introduction,” the mechanism of action of tacrolimus is known. Tacrolimus-induced inhibition of the calcineurin pathway inhibits the activity of NFAT, a transcription factor that regulates *IFN*γ gene expression (48, 49). Our results demonstrate that this tacrolimus-induced suppression of IFNγ protein production is independent of changes in DNA methylation of *IFN*γ.

Mycophenolic acid did not affect the percentage of IFNγ producing memory cells in our experiments but the results reported in literature vary. He et al. (41) reported that MPA inhibited IFNγ production in CD4^+^ T cells after α-CD3/CD28 stimulation. Whereas Egli et al. (50) did not find a strong decrease in IFNγ production after adding MPA to CMV-stimulated PBMCs. In both studies, IFNγ concentration was measured in the culture supernatant, and such concentration is strongly related to the number of cells present. Since proliferation decreases under the influence of MPA (18, 51), cytokine production should be corrected for cell numbers as we did by measuring intracellular IFNγ. In addition, Egli et al. (50) did not measure T-cell specific IFNγ production and since NK cells are also capable of producing IFNγ this may have influenced their results. These experimental differences could explain the difference between our findings and the results reported in literature.

Here, we focused on the *IFN*γ gene promoter to study differences in DNA methylation. Possibly, immunosuppressive drugs have much stronger effects on DNA methylation of other genes or even at intergenic regions (12). To find the most affected regions, a genome-wide methylation study could be performed. Due to the explorative nature of this study, a genome-wide approach was outside the scope of this paper.

The findings presented here demonstrate that *IFN*γ DNA methylation in T cells was not affected in the same manner by tacrolimus and MPA and therefore we conclude that these immunosuppressive drugs differentially affect *IFN*γ DNA methylation in CMV seropositive individuals. Our study also shows that naive and memory T cells did not only have distinct DNA methylation profiles, but also that they were not affected equally by the immunosuppressive drugs studied. These findings may be of significance for future research into the efficacy of immunosuppressive drugs. Knowledge on the effect of immunosuppressive drugs on DNA methylation of T-cell effector genes and thereby T-cell function could optimize the treatment regimen. When developing and testing immunosuppressive drugs, we recommend to include DNA methylation studies thereby improving our understanding of their effect on the function of patients’ immune cells.

## Ethics Statement

This study was carried out in accordance with the recommendations of the biobank protocol (MEC-2010-022) with written informed consent from all subjects. All subjects gave written informed consent in accordance with the Declaration of Helsinki. The protocol was approved by the local ethics committee (METC).

## Author Contributions

FP contributed to designing, performing, and analyzing the experiments, interpreting the results, and writing of the manuscript. AP performed the experiments. LH provided the analytical tools. MB reviewed the manuscript. KB and CB both contributed to designing the experiments, interpreting the results, and writing of the manuscript.

## Conflict of Interest Statement

The authors declare that the research was conducted in the absence of any commercial or financial relationships that could be construed as a potential conflict of interest.
