# Supplementary material for: Interferon-Gamma DNA Methylation Is Affected by Mycophenolic Acid but Not by Tacrolimus after T-Cell Activation
Source: Front Immunol. 2017 Jul 12;8:822. doi: 10.3389/fimmu.2017.00822 (PMC5506181; doi:10.3389/fimmu.2017.00822)
Supplement: Supplementary file 1 [file Data_Sheet_1.PDF]

Supplementary Material

Interferon-gamma DNA Methylation is Affected by MPA but not by Tacrolimus after T-cell Activation

FS Peters[1*], AMA Peeters[1], LJ Hofland[2], MGH Betjes[1], K Boer[1], CC Baan[1]

[1]Nephrology and Transplantation, Department of Internal Medicine, Erasmus MC, Erasmus University Medical Center Rotterdam, The Netherlands

[2]Endocrinology ,Department of Internal Medicine, Erasmus MC, Erasmus University Medical Center Rotterdam, The Netherlands

***Corresponding author: FS Peters, f.s.peters@erasmusmc.nl**

Supplementary Data

Supplementary Figure 1: Proliferation of total T cells presented as the median of cells per well in time. Stimulation (n=9), decitabine (n=7), tacrolimus (n=5) and MPA (n=4). 50,000 cells were stimulated at day 0 and the cells were counted at day 1 and 3 after stimulation with conventional light microscopy after staining the cells with Trypan Blue.

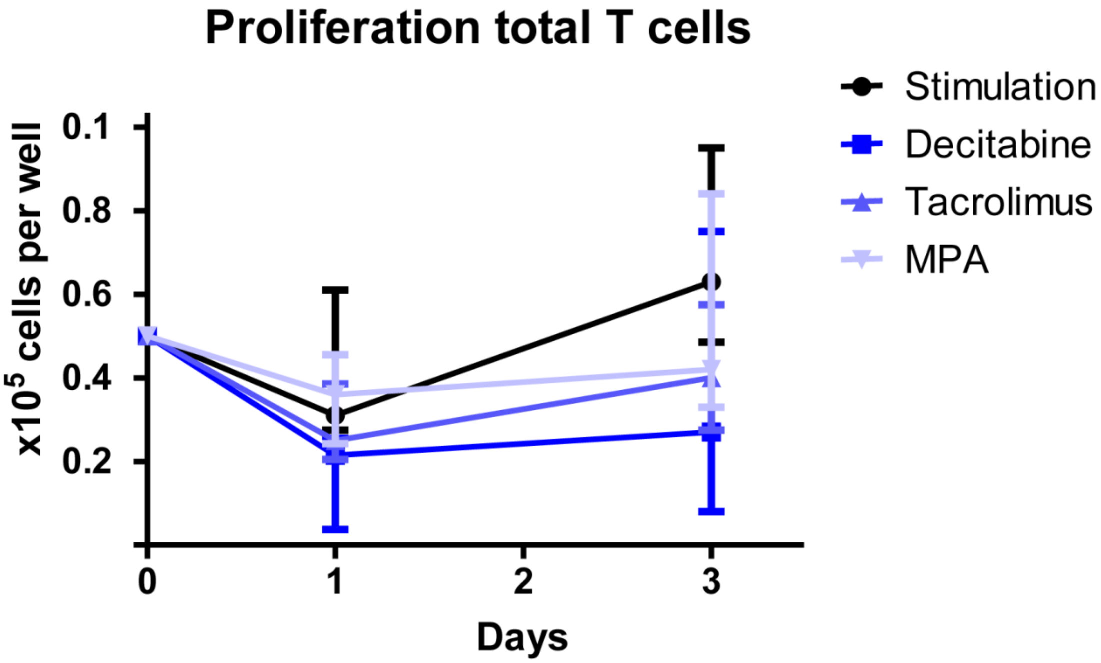

Supplementary Figure 2: Median percentages of CD4+ and CD8+ populations within the CD3+ cells of A) the naive start population (CCR7+CD45RO-) in the presence and absence of tacrolimus or MPA and B) the memory start population CD45RO+ and CCR7-CD45RO-) in the presence and absence of tacrolimus or MPA.

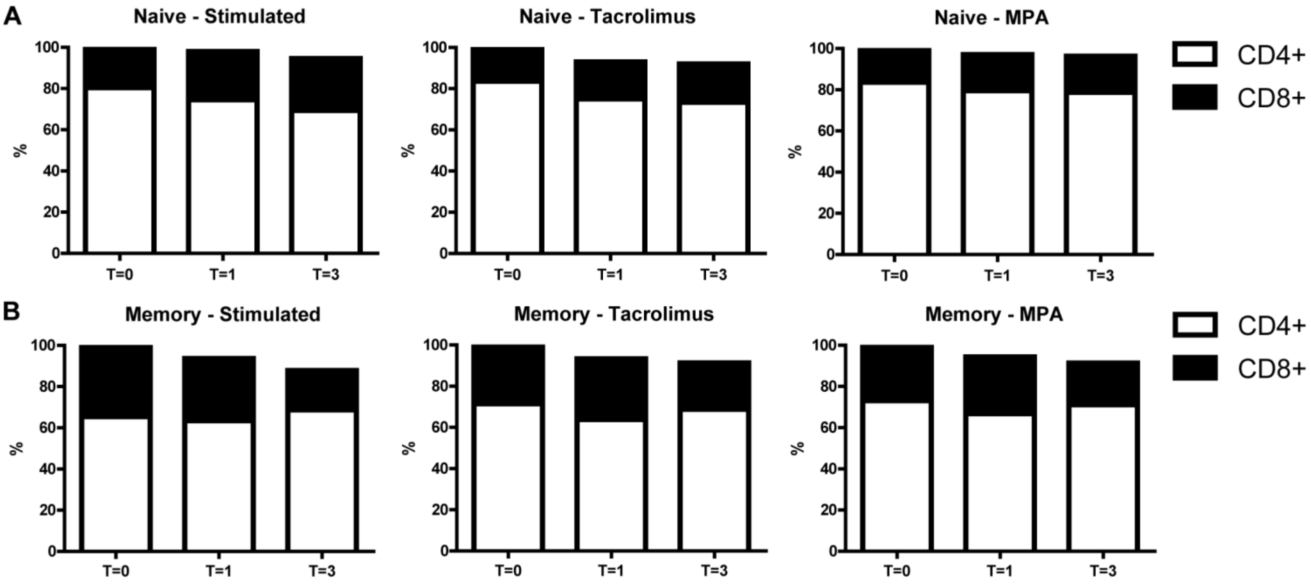

frontiers
